# Supplementary material for: Succinate Is an Inflammation-Induced Immunoregulatory Metabolite in Macrophages
Source: Metabolites. 2020 Sep 15;10(9):372. doi: 10.3390/metabo10090372 (PMC7569821; doi:10.3390/metabo10090372)
Supplement: Supplementary file 1 [file metabolites-10-00372-s001.pdf]

Supplementary Materials:

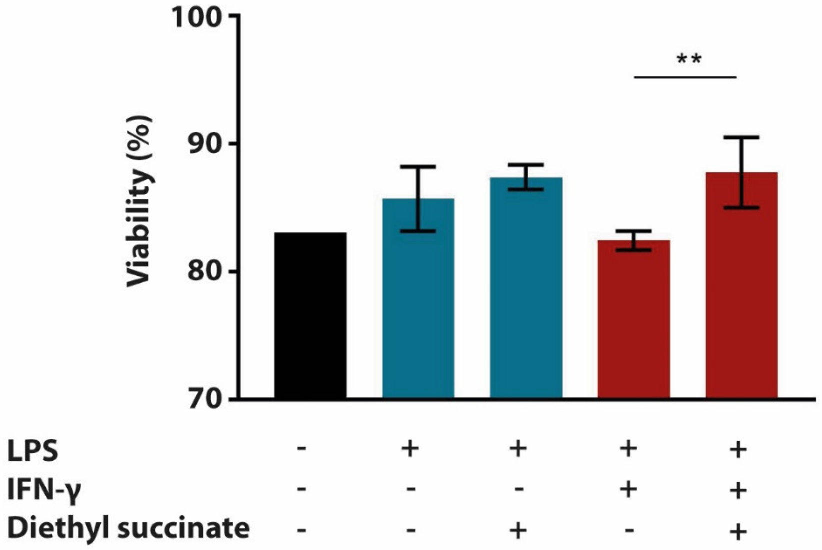

Figure S1. Cell-permeable succinate increases viability of inflammatory BMDMs. BMDMs were pre-treated with diethyl succinate (5 mM) 1 hour prior to LPS (100 ng/mL) or LPS + IFN-γ (10 ng/mL + 10 U/mL, respectively) stimulation for 24 hours. Cells were subsequently stained with fixable viability dye. Stained cells were then read by a BD LSRFortessa X-20 and cell biomarker surface expression was analysed in FCSExpress 7. All data are in quadruplicates represented by mean ± SD and are representative of three independent experiments. *$p \leq 0.05$, **$p \leq 0.01$, ***$p \leq 0.001$, ****$p \leq 0.0001$.

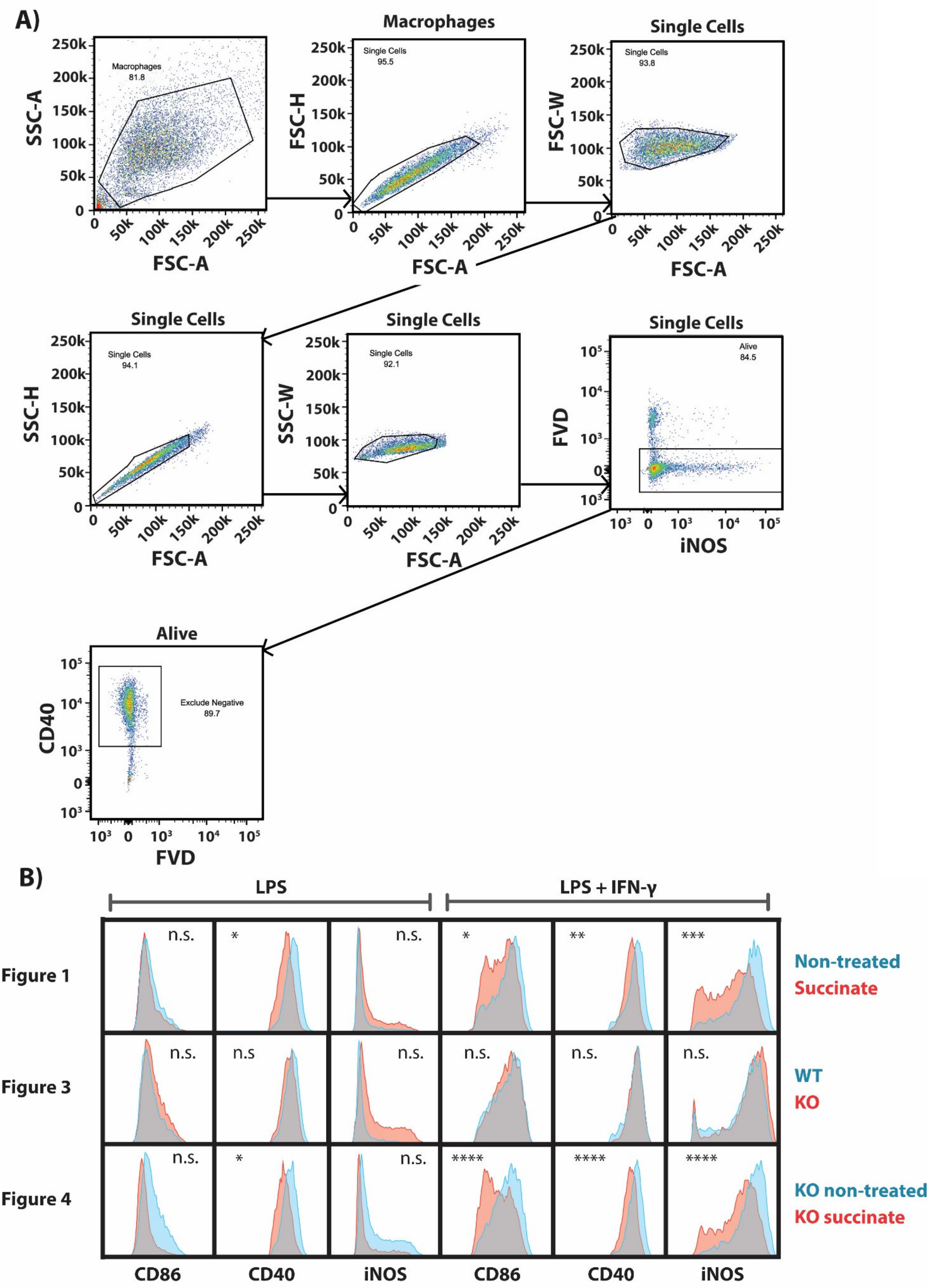

Figure S2. Gating strategy and overlay plots of macrophage marker expression. (**A**) Here we supply the gating strategy used to analyse cell marker expression for all figures in the manuscript. The gate

of "exclude negative" was drawn to remove cells negative for all markers used and subsequently used for generating expression results. (**B**) representative overlay plots of triplicate samples stained for CD86, CD40 and iNOS are displayed with annotations of which figures from the manuscript they correspond to. All overlays are normalised to the mode on the y-axis in order to improve peak comparisons. Sample conditions for each figure are shown in blue (control) or red (experimental sample). The left half of the panel are LPS and the right are LPS + IFN-γ treated macrophages. Significance are shown in each overlay where *$p \leq 0.05$, **$p \leq 0.01$, ***$p \leq 0.001$, ****$p \leq 0.0001$.

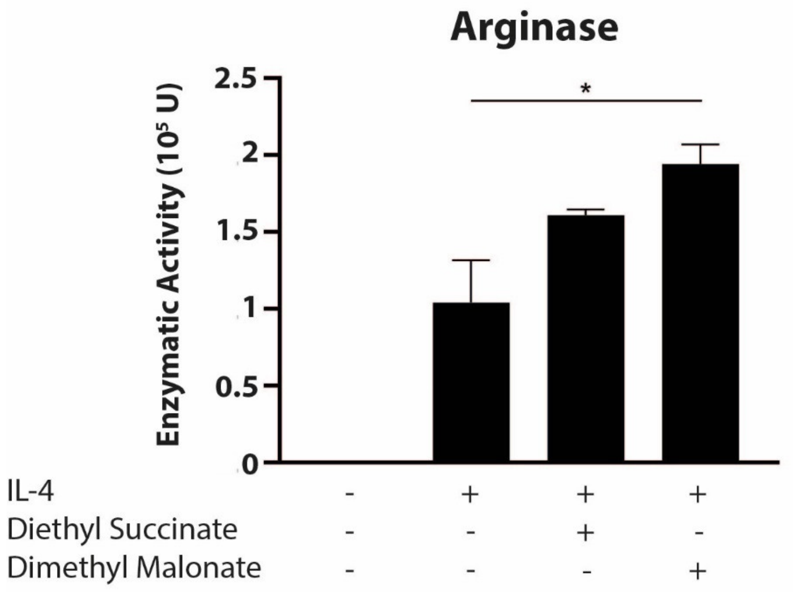

Figure S3. Arginase activity in IL-4-stimulated macrophages is enhanced by cell-permeable succinate and malonate**.** BMDMs were pre-treated with either diethyl succinate (5 mM) or dimethyl malonate (10 mM) 1 hour prior to IL-4 (20 ng/mL) stimulation for 24 hours. Cells were subsequently assessed for arginase activity. All data are in duplicate represented by mean ± SD. *$p \leq 0.05$, **$p \leq 0.01$, ***$p \leq 0.001$, ****$p \leq 0.0001$.

Table S1. List of used antibodies, dyes and chemicals.

**Antibodies**		
**Marker/Substrate**	**Clone**	**Supplier**
Mouse iNOS-PE	CXNFT	eBioscience
Mouse CD40-APC	3/23	BD BioSciences
Mouse CD86-BV510	GL-1	BioLegend
Mouse CD301-PerCP/Cy5.5	LOM-14	BioLegend
Mouse CD301b-PE/Cy7	URA-1	BioLegend
Mouse CD16/CD32 (Fc-block)	93	eBioscience
**Dyes**		
Fixable Viability Dye-eFluor™ 780	-	eBioscience
**Chemicals**		
Diethyl succinate	-	Sigma Aldrich
Disodium succinate	-	Sigma Aldrich
Dimethyl malonate	-	Sigma Aldrich
